# Unique nonstructural proteins of Pneumonia Virus of Mice (PVM) promote degradation of interferon (IFN) pathway components and IFN-stimulated gene proteins

**DOI:** 10.1038/srep38139

**Published:** 2016-12-01

**Authors:** Jayeeta Dhar, Sailen Barik

**Affiliations:** 1 Department of Biological, Geological and Environmental Sciences, and Centre for Gene Regulation in Health and Disease, Cleveland State University, 2121 Euclid Avenue, Cleveland, Ohio 44115, USA.

## Abstract

Pneumonia Virus of Mice (PVM) is the only virus that shares the *Pneumovirus* genus of the *Paramyxoviridae* family with Respiratory Syncytial Virus (RSV). A deadly mouse pathogen, PVM has the potential to serve as a robust animal model of RSV infection, since human RSV does not fully replicate the human pathology in mice. Like RSV, PVM also encodes two nonstructural proteins that have been implicated to suppress the IFN pathway, but surprisingly, they exhibit no sequence similarity with their RSV equivalents. The molecular mechanism of PVM NS function, therefore, remains unknown. Here, we show that recombinant PVM NS proteins degrade the mouse counterparts of the IFN pathway components. Proteasomal degradation appears to be mediated by ubiquitination promoted by PVM NS proteins. Interestingly, NS proteins of PVM lowered the levels of several ISG (IFN-stimulated gene) proteins as well. These results provide a molecular foundation for the mechanisms by which PVM efficiently subverts the IFN response of the murine cell. They also reveal that in spite of their high sequence dissimilarity, the two pneumoviral NS proteins are functionally and mechanistically similar.

Pneumonia Virus of Mice (PVM) and Respiratory Syncytial Virus (RSV) are the only members of the *Pneumovirus* genus in the *Paramyxoviridae* family^1^,^2^,^3^. While human RSV is highly pathogenic in humans, especially in the young and the elderly, PVM causes high morbidity and mortality in mice^1^,^2^,^3^,^4^,^5^. The nonsegmented negative-strand RNA genomes of both viruses encode two promoter-proximal genes that code for nonstructural proteins 1 and 2 (NS1 and NS2). The NS proteins of RSV have been studied in some detail and shown to circumvent the host innate immune system by preventing the induction of type I interferons (IFNs) as well as IFN-induced antiviral responses^6^,^7^,^8^,^9^,^10^,^11^,^12^,^13^,^14^,^15^,^16^,^17^,^18^,^19^,^20^,^21^, thus allowing a more robust replication of the virus, leading to the severe respiratory disease that characterizes RSV infection. Recent studies have indicated that the two NS proteins of PVM also possess IFN evasion function, although the molecular mechanism remains unknown^22^,^23^.

Induction of cellular IFN by RNA viruses primarily involves activation of a cytoplasmic RNA sensor of the RIG-I family^24^,^25^. The CARD sequences of the activated RIG-I interact with the CARD-like domain of the mitochondrial protein, MAVS, which then activates TRAF3 which in turn activates two downstream kinases, IKKε and TBK1^26^. These kinases phosphorylate and activate IRF3 and IRF7, the transcription factors that are essential for the induction of type I IFN genes^26^,^27^. The liberated type I IFN binds to its cognate receptors, triggering the IFN response pathway, in which transcription factors STAT1 and STAT2, along with IRF9 forms a tripartite complex that transcriptionally induce a large family of IFN-responsive genes^28^,^29^. Several genes of this family have been shown to code for antiviral proteins that confer resistance to the virus^30^,^31^.

In previous studies, we and others reported that the NS proteins of human RSV, expressed in human cells, degrade multiple proteins of the IFN pathways, notably RIG-I and IRF3 of the IFN induction pathway, and the STAT2 protein of the IFN response pathway^9^,^18^,^19^,^21^. Here, we report that the NS proteins of PVM, even though their primary structures bear no similarity to the RSV counterparts (Supplementary Fig. 1), target common IFN pathway components in mouse cells. Moreover, the PVM NS proteins, like the RSV counterparts, appear to promote the degradation of the IFN components by a ubiquitin-based proteasomal mechanism. Thus, the NS proteins of the two pneumoviruses, in spite of their sequence divergence, have evolved to be functionally and mechanistically homologous.

## Results

### Sequence dissimilarity between RSV and PVM NS proteins

In an effort to understand how PVM NS proteins may function, we compared their predicted primary structures with those of the RSV NS proteins that are established IFN suppressors. However, the NS proteins of the two viruses were highly dissimilar with no common sequence motifs (Supplementary Fig. 1). Even the C-terminal DLNP tetrapeptide, shared between RSV NS1 and RSV NS2 proteins, is absent in both the NS proteins of PVM. Thus, we reasoned that the mechanism by which PVM NS proteins suppress IFN may differ from that of the RSV proteins and, hence, must be studied independently.

### PVM NS proteins lower the levels of mouse RIG-I, IRF3 and STAT2

To understand the role of the PVM NS proteins in IFN evasion, we adopted the overexpression approach that has been successfully used for RSV NS proteins in the recent past. We cloned codon-optimized NS sequences in pCAGGS vector so that the proteins are expressed with an N-terminal FLAG tag. In denaturing SDS-PAGE, both NS1 and NS2 ran true to their predicted molecular weights, respectively 13 kDa and 18 kDa. We then expressed these recombinants together with the mouse homologs of various IFN pathway proteins by transient co-transfection in mouse embryonic fibroblast (MEF) cells. The steady-state levels of the IFN pathway proteins were then measured by immunoblot. We note here that in all our transfection experiments the recombinant NS2 protein is produced more abundantly than the NS1 protein, the reason for which is unknown.

**Figure 1:**
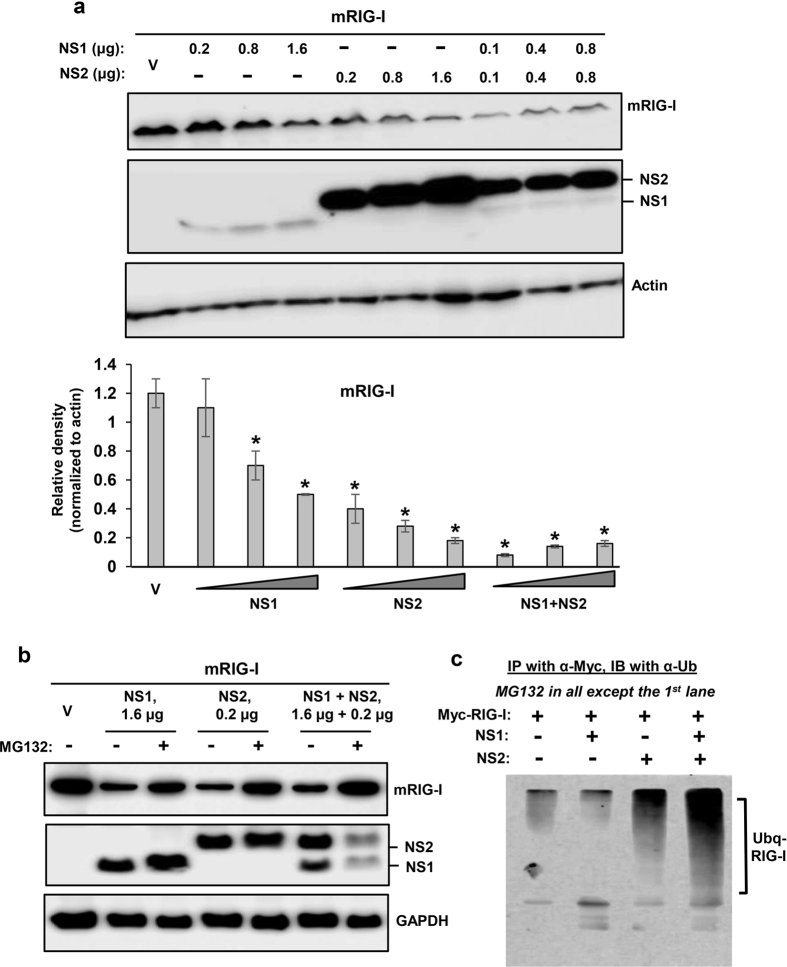
Proteasomal degradation of mouse RIG-I by PVM NS proteins. MEF cells in 24-well plates were transiently transfected with 0.4 μg Myc-FLAG-mRIG-I expression plasmid and indicated amounts of pCAGGS-FLAG-NS plasmids. Cells were processed 24 h later for immunoblot (Western) to detect the proteins as shown. Myc-FLAG-RIG-I was detected by FLAG antibody. Note that our NS1-expressing plasmid consistently produces less protein than the NS2-expression plasmid, for reasons that are unclear. Cells were cultured in the (**a**) absence or (**b**) presence of MG132 (10 μM, added in the medium at 8 h after plasmid transfection), as shown. Actin and GAPDH are loading controls. Densitometry and plot of RIG-I band intensities were performed as described under Methods. V indicates transfection with empty (no-NS) pCAGGS vector. For ubiquitination analysis (**c**), cells were cultured similarly, immunoprecipitation was performed as described^18^,^19^ and the precipitate subjected to immunoblot using pan anti-Ub antibody. The ubiquitinated forms of RIG-I are indicated.

**Figure 2:**
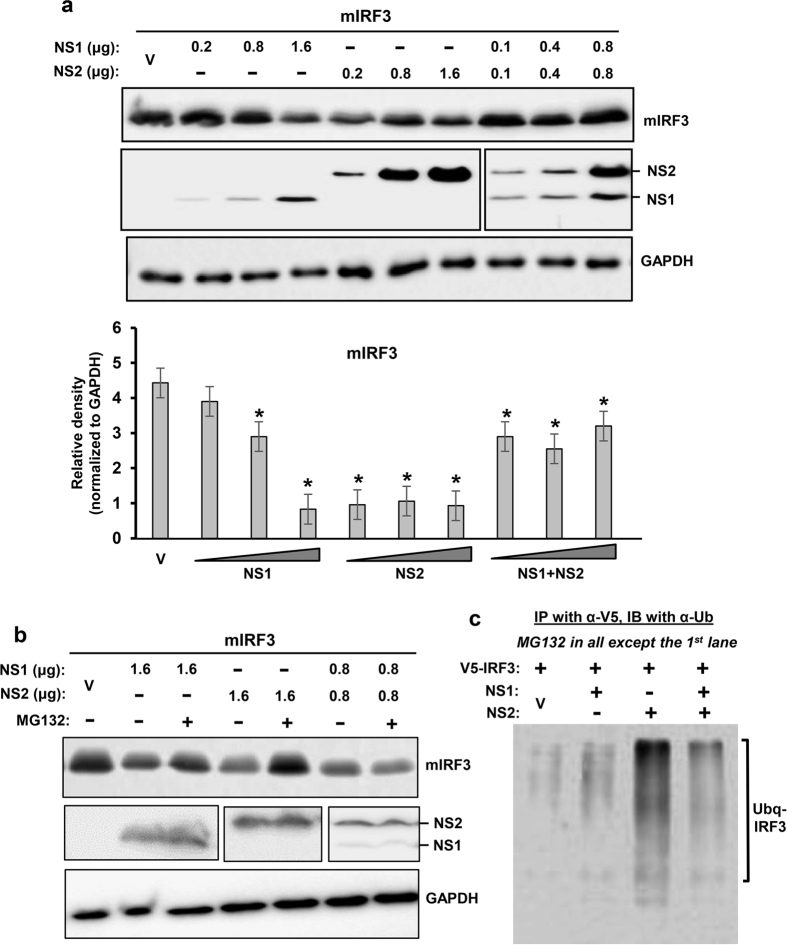
Proteasomal degradation of mouse IRF3 by PVM NS proteins. These experiments were designed and performed essentially as in Fig. 1, with 0.4 μg V5-mIRF3 replacing the RIG-I expression plasmid. Immunoblot shows the levels of the indicated proteins. As in Fig. 1, cells were cultured in the (**a**) absence or (**b**) presence of MG132. GAPDH is loading control. Densitometry and plot of IRF3 band intensities were performed as described under Methods. Ubiquitination analysis (**c**) was also performed essentially as in Fig. 1c, and the ubiquitinated forms of IRF3 are indicated.

**Figure 3:**
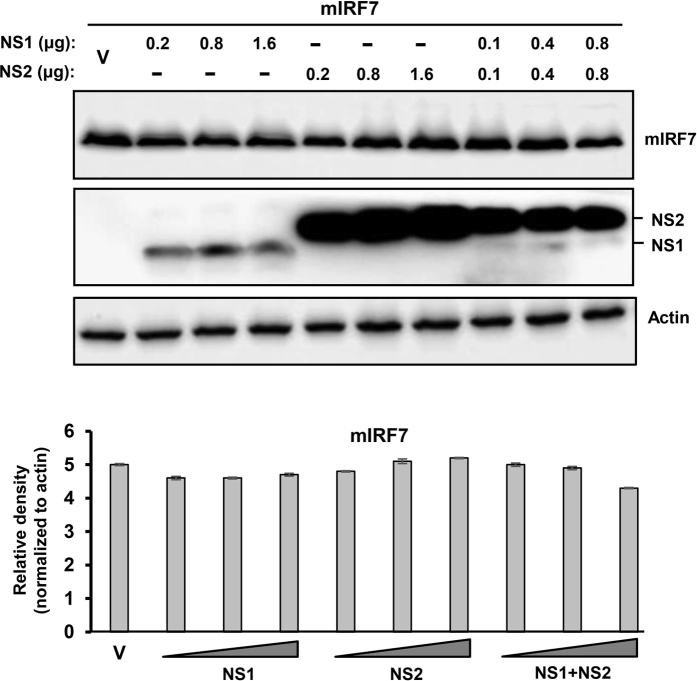
Lack of effect of PVM NS proteins on mouse IRF7. These experiments were designed and performed essentially as in Fig. 1, with 0.4 μg FLAG-mIRF7 replacing the RIG-I expression plasmid. Immunoblot shows the levels of the indicated proteins, and the densitometry of IRF7 bands is presented below. Actin was loading control.

We first tested the PVM NS proteins against mouse RIG-I (mRIG-I), the virus-proximal member of the IFN induction pathway, and found that both NS proteins were effective in lowering RIG-I level (Fig. 1a), although we consider NS1 to be more effective, because in this particular experiment recombinant NS1 was expressed at considerably lower amounts than NS2. Restoration of RIG-I levels by MG132, a proteasome inhibitor (Fig. 1b), suggested that the NS proteins promoted RIG-I degradation by recruiting the cellular proteasome. We then examined the downstream signalling molecules, and found that IRF3 was also targeted for degradation by PVM NS (Fig. 2b), although not as efficiently as RIG-I. When normalized against GAPDH, and at comparable amounts of NS proteins (e.g. at 1.6 μg NS1 plasmid and 0.2 μg NS2 plasmid), NS2 appeared to be more effective than NS1. When expressed together, NS1 not only failed to enhance NS2 activity but appeared to have an inhibitory effect. As with RIG-I, IRF3 levels were also substantially restored by MG132 (Fig. 2b). If the degradation of RIG-I and IRF3 is due to proteasomes, one would expect to detect their ubiquitinated forms in the presence of MG132. Indeed, slower-migrating forms of both proteins could be detected in immunoblot, using ubiquitin (Ub) antibody (Figs 1c and 2c). In contrast to these two substrates, IRF7 was not at all targeted by either NS1 or NS2 (Fig. 3), demonstrating specificity of NS action.

**Figure 4:**
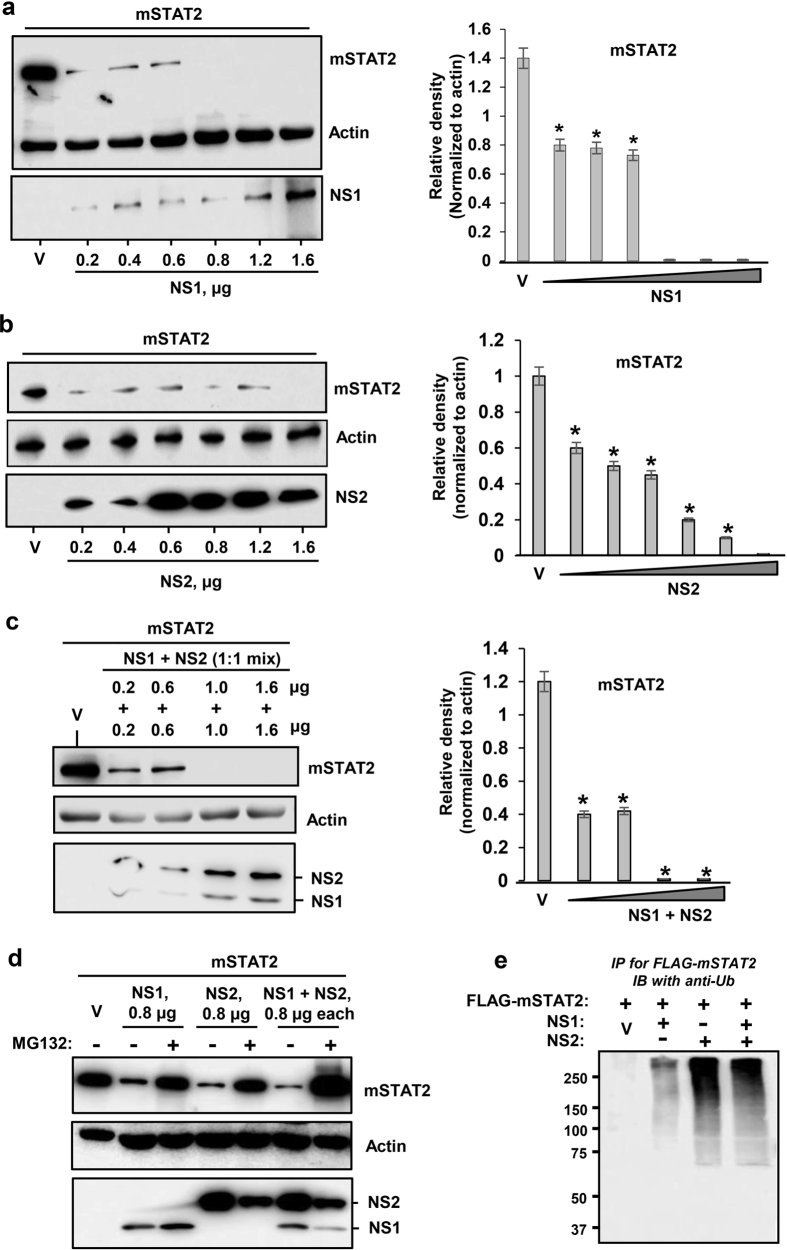
Degradation of mouse STAT2 by PVM NS proteins. Transient transfection was performed as in Fig. 1 to express FLAG-mSTAT2 along with (**a**) NS1 protein, (**b**) NS2 protein or (**c**) both. Right panels show densitometric plot of the corresponding substrate bands on the left. Strong reduction of STAT2 is observed in all three panels (**a**,**b**,**c**). Actin is loading control. V indicates 0.4 μg vector (pCAGGS, no NS). (**d**,**e**) PVM NS proteins promote ubiquitination and proteasomal degradation of mSTAT2. These experiments are essentially similar to Fig. 1c, FLAG-mSTAT2 being the substrate here. Protection of STAT2 by MG132 (**d**) and the ubiquitinated form of STAT2 (**e**) are demonstrated. Where shown, ‘V’ indicates vector-only (no NS), and actin is the loading control.

In the IFN response pathway, STAT2 was previously found to be the sole substrate of the RSV NS proteins; therefore, we studied the effect of PVM NS proteins on recombinant mouse STAT2 in some detail. Indeed, both NS proteins of PVM lowered mSTAT2 levels with high efficiency (Fig. 4a–c). The loss could be inhibited by MG132 (Fig. 4d), and ubiquitinated STAT2 could also be detected under these conditions (Fig. 4e), again suggesting a ubiquitin-dependent proteasomal mechanism.

To rule out an effect of NS on mRNA, we quantified the steady-state mRNA levels of recombinant mRIG-I, mIRF3, mSTAT2 as well as mIRF7 in PVM NS-transfected and untransfected MEF cells by qRT-PCR, and no difference was observed (data not shown).

### PVM lowers the steady-state levels of IFN-pathway proteins

**Figure 5:**
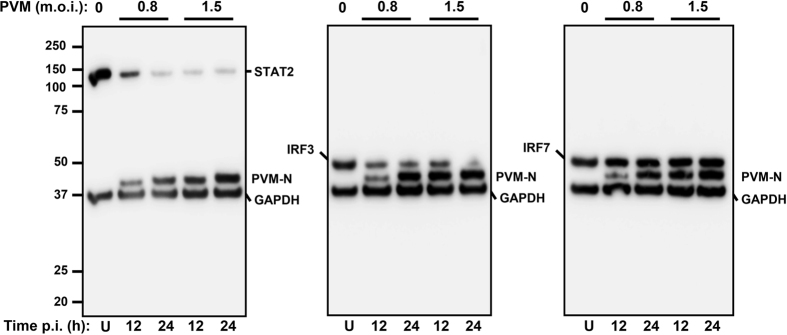
Infection by PVM promotes loss of IFN pathway proteins. MEF cells were transfected with FLAG-STAT2, V5-IRF3 or Myc-FLAG-IRF7 plasmid as described before, and then infected with freshly grown PVM at the specified m.o.i. At 12 h and 24 h post-infection cells were analysed for the indicated proteins by immunoblot with a mixture of the corresponding antibodies. Note the reduction of STAT2 and IRF3, but not IRF7 (detected by FLAG antibody), by virus infection. Replication of the virus is confirmed by expression of the N protein in an inoculum- and time-dependent manner. U = uninfected cells.

Since the experiments described thus far used recombinant NS proteins, we needed to validate the findings in PVM-infected cells. Here, we used RIG-I and IRF3 as representative NS-sensitive targets and IRF7 as the resistant one. MEF cells were transiently transfected with these recombinant substrate plasmids as before and cells were infected with PVM at two different m.o.i. (0.8 and 1.5). The amount of PVM nucleocapsid protein, N, increased with time of infection, especially appreciable at the lower m.o.i. At different time points of infection, samples were analysed by immunoblot. Results (Fig. 5) documented loss of IRF3 and STAT2 in a viral dose-dependent and time-dependent manner, whereas IRF7 remained unaffected. Thus, as with recombinant NS proteins, PVM also promoted degradation of STAT2 and IRF3.

### Suppression of IFN by PVM NS proteins in luciferase reporter assay

**Figure 6:**
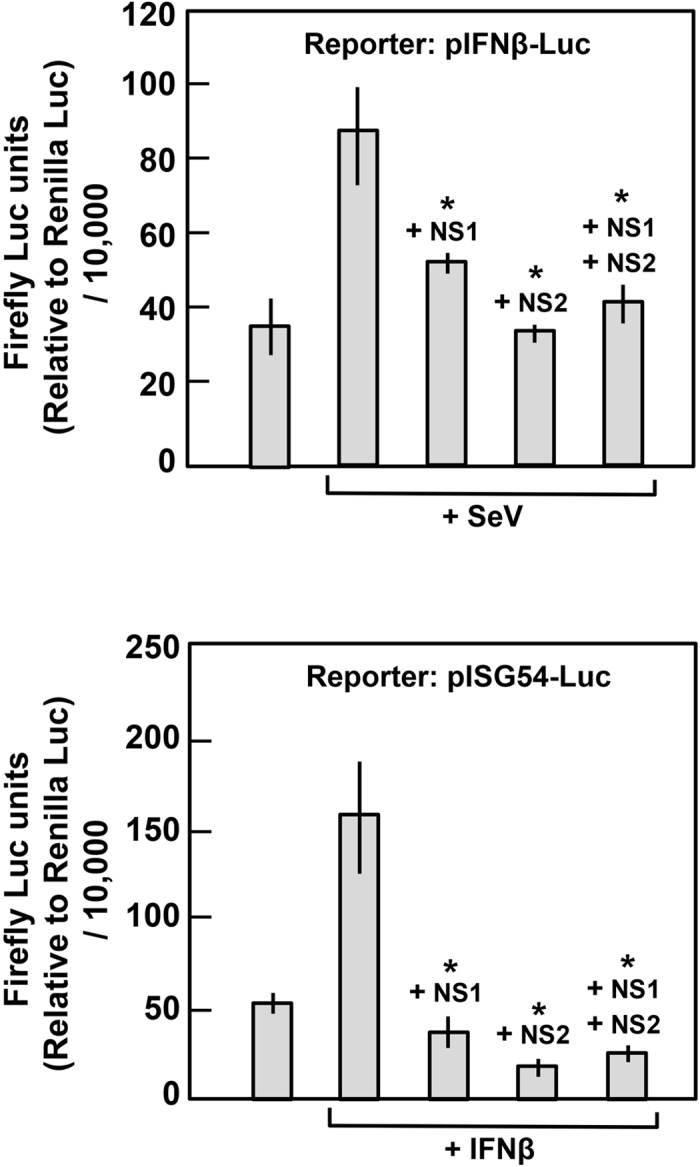
Functional IFN suppression assay of PVM NS proteins. MEF cells in each of 24-well plates were transfected with 1.6 μg NS1 plasmid or 0.2 μg NS2 plasmid or a mixture of the two, along with the indicated reporter firefly Luc plasmids for IFN induction (Top) or IFN response assay (Bottom). In addition, at 6 h post-transfection, Sendai virus (SeV) at an approximate mo.i. of 2.0 was added to induce the IFN promoter (Top) and recombinant universal type I IFN (human IFN-alpha A/D-BglII; PBL Sciences) (1,000 U/ml) was added to induce the ISG54 promoter (Bottom). All cells were also transfected with 0.05 μg of CMV-Renilla Luc plasmid for transfection control. Cell extracts made at 24 h post transfection were assayed for both luciferases using a dual-luciferase assay kit (Promega). Relative firefly Luc units were expressed after normalization against Renilla Luc. Asterisk denotes significant reduction (*p* < 0.05) of Luc expression by NS proteins.

To test the suppressive effect of the NS proteins in a functional assay, we used established reporter plasmids in which transcriptional expression of the luciferase (Luc) gene is driven by the IFN-β gene promoter or the ISG54 promoter, which provide a measure of IFN induction or response pathway, respectively. MEF cells were transiently transfected with these Luc plasmids along with PVM NS-expressing plasmids under appropriate conditions and Luc levels were measured. Indeed, both NS1 and NS2 were found to suppress Luc expression from both reporters (Fig. 6), consistent with their ability to reduce members of both IFN induction and response pathways.

### Reduction of specific ISG levels by PVM NS proteins

**Figure 7:**
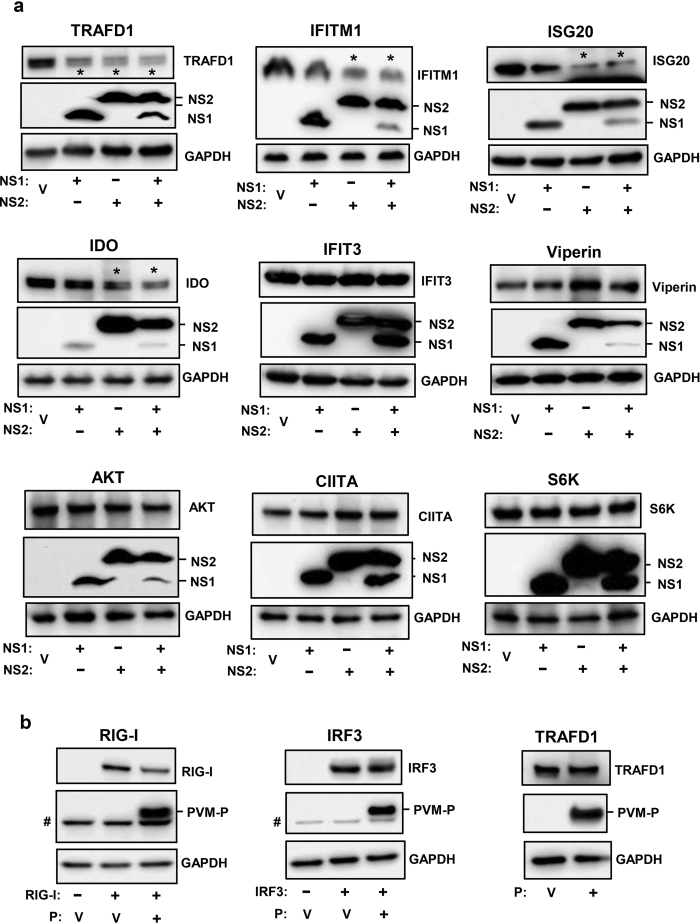
Degradation of specific ISGs by PVM NS proteins but not by PVM P protein. (**a**) Tet-induced FLAG-ISG-expressing cells were transfected as described in Methods with 1.6 μg FLAG-NS1 or 0.2 μg FLAG-NS2 plasmid or both, and cells were harvested for immunoblot analysis using FLAG antibody as primary antibody to detect the ISG and NS proteins. Four ISGs (TRAFD1, IFITM1, ISG20, IDO), affected by NS proteins (of various combinations), are shown first, followed by two ISGs (IFIT3, Viperin) that are representative of many that were not affected. Three proteins, not related to IFN pathways (AKT, CIITA, S6K), were also not affected. (**b**) MEF cells were transfected with 1.6 μg of FLAG-RIG-I or V5-IRF3 plasmids (as in Figs 1 and 2, respectively), along with FLAG-PVM-P (1.6 μg) plasmid where indicated. For TRAFD1, the Tet-inducible clone was induced with Tet (as in panel ‘a’). A nonspecific band of MEF origin, migrating just below the P band, is marked by #; it is not seen in the HEK293 cells. In all panels, V indicates transfection with empty pCAGGS vector (i.e. no NS or P). The IFITM1 gel was 12% polyacrylamide; all others were 4–20% gradient.

It is currently unknown which ISG(s) can inhibit PVM; however, we have observed that RSV NS1 protein promotes degradation of OASL (2′-5′-Oligoadenylate Synthetase Like), an ISG that is antagonistic to RSV replication^32^,^33^, suggesting that the IFN suppressors of a virus may have evolved to counteract specific host functions that directly inhibit the virus. Since we established a degradative assay for the PVM NS proteins, we moved on to determine if an ISG is also targeted by these proteins. To this end, we used a panel of available HEK293 cell lines, in which specific ISGs are inducible by tetracycline (Tet)^34^,^35^. Although these cells expressed human ISGs in human cell background, they allowed us to interrogate several well-known ISGs. We transiently transfected these Tet-induced ISG-expressing cells with PVM NS plasmids (or with empty pCAGGS vector), and measured the steady-state levels of FLAG-tagged ISG proteins in these cells by immunoblot. To summarize this set of results (Fig. 7a), significant reduction was observed for TRAFD1, IFITM1 and ISG20 with various combinations of NS1, NS2 or both. IDO was affected modestly, and IFIT3 and viperin were unaffected. Three proteins, unrelated to IFN pathway, were also tested; they were AKT (also known as Protein Kinase B), CIITA (Class II Major Histocompatibility Complex Transactivator) and S6K (ribosomal S6 protein kinase), all of which were also unaffected by NS proteins. Overall, these results extend the substrate repertoire of the pneumoviral nonstructural proteins to include specific members of the ISG family.

Even though the NS proteins degraded a small subset of the host proteins (i.e., did not degrade many others), we felt that another PVM protein was worth testing for further confirmation of NS specificity. We thus expressed the phosphoprotein (P) gene of PVM from the same pCAGGS vector, optimized its expression, and then tested its ability to degrade three representative substrates that were affected by the NS proteins, namely RIG-I, IRF3 and TRAFD1. Results (Fig. 7b) showed that all three were essentially unaffected by PVM P, although the small decrease of RIG-I was consistent (82% and 78% of the PVM P-untreated value in two experiments). Thus, we conclude that degradative effect of NS proteins is a specific property of these viral proteins, which is not replicated by the P protein.

### Interaction between IFN pathway proteins and PVM NS

**Figure 8:**
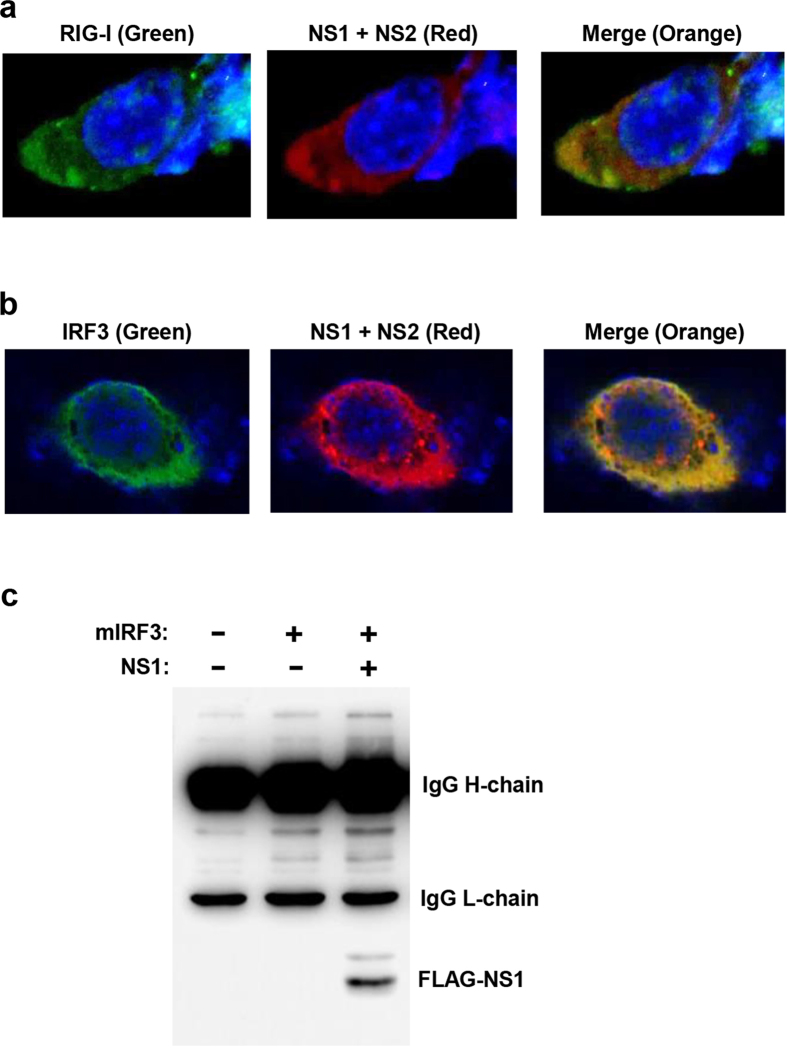
Interaction of NS proteins with their cellular substrates. (**a**,**b**) Immunofluorescence microscopy, performed as described in Methods, show co-localization of PVM NS proteins (Red) with substrates RIG-I or IRF3 (Green), indicated by orange colours in the merged panel. Nuclei are DAPI-stained (Blue). (**c**) Immunoprecipitation (IP) of V5-IRF3 using mouse anti-V5 antibody, followed by immunoblotting with mouse anti-FLAG antibody (which provides cleaner background than the corresponding rabbit antibody), detected FLAG-NS1 as shown. The dark upper bands are heavy and light chains of the immunoprecipitating V5 antibody, detected by the secondary antibody. In both microscopy and IP, MG132 was added to the cell culture to stabilize the substrates, so that the substrate-NS complexes could be detected.

In RSV, the NS proteins were found to co-localize with their substrates in the cellular cytoplasm^8^,^19^,^21^. Since co-localization can serve as a mechanism that facilitates specific degradation of these substrates by NS proteins, we tested whether the PVM NS proteins also interacts and/or co-localise with their substrates. We tested two substrates, RIG-I and IRF3, co-expressed with recombinant FLAG-tagged NS1 and NS2 of PVM, studied their localization by immunofluorescence confocal microscopy, whereby both substrates were found to co-localize with NS proteins in the cytoplasm, with a perinuclear preference (Fig. 8a,b). In an independent procedure, we also demonstrated NS1-IRF3 interaction in pulldown experiment, whereby we could detect FLAG-NS1 in the immunoprecipitate of V5-IRF3 (Fig. 8c). Together, both techniques demonstrated association of PVM NS proteins with these cellular substrates.

## Discussion

In this study, we demonstrate that despite their highly dissimilar amino acid sequences, the two nonstructural (NS) proteins of the pneumoviruses, RSV and PVM, promote degradation of multiple, albeit specific, members of the IFN pathway using the host cell’s ubiquitin-proteasome system. We also show for the first time that the NS proteins of PVM promote degradation of selected murine ISGs, the most distal arm of the type I IFN pathway. Recent studies, including ours, have shown that at least two ISGs can act as antivirals against RSV; they are viperin and OASL^32^,^33^,^36^. We also showed that RSV NS1 could degrade OASL, allowing better RSV growth^33^. It is reasonable to assume that the NS proteins of both viruses are capable of inhibiting or degrading many more members of the ISG family that need to be identified. Indeed, there are a few hundred ISGs (the exact number being unknown), the antiviral function of most of which is yet to be characterized. It will be interesting to determine the full repertoire of ISGs that may inhibit RSV and PVM and how the two NS proteins counteract them to allow optimal viral replication and the resultant pathogenesis.

In RSV, NS1 and NS2 form homo- and hetero-dimers, and also exhibit a certain degree of substrate preference^18^,^19^,^21^. We have not yet tested the interactions between NS1 and NS2 of PVM, but they also appear to degrade various substrates to different extents. For example, STAT2 is degraded by both NS with equal efficiency (Fig. 4), and so is TRAFD1 (Fig. 7a); however, both IFITM1 and ISG20 are preferentially degraded by NS2 (Fig. 7a). In the case of IRF3, the relatively nonfunctional NS1 appeared to inhibit the functional NS2, which may be due to the formation of NS1-NS2 heterodimers, sequestering the functional NS2 into an inactive complex that can no longer recognize IRF3. Clearly, the mechanism of substrate specificity and the interactive property of the two pneumoviral NS proteins should shed important light on their structure and function.

In this regard, the sequence dissimilarity between RSV and PVM NS proteins is intriguing, particularly as they ultimately have similar functions, i.e. suppression of IFN through the degradation of several IFN pathway proteins. The proposed SOCS box of these proteins also remains elusive. The canonical cellular Elongin-Cullin-SOCS box ubiquitin ligase is composed of Elongin B (EloB), Elongin C (EloC), Cullin 5 (Cul5), Rbx2, and a SOCS box protein that acts as a scaffold, linking the E3 ligase complex with its substrates^37^. The SOCS box, a region of ~35 amino acids, contains a BC box, which binds EloB and EloC (EloBC), and the Cullin box, which recruits either Cul5 or Cul2^38^. Although multiple paralogs of Rbx and Cullin have been recognized, the exact subunit responsible for the substrate specificity of the E3 ligase remains an unsolved mystery. This is particularly evident for the NS proteins that promote the ubiquitination of multiple yet selective substrates. RSV NS1, for example, recruits EloC, Cul2, and an Rbx subunit, all of which were essential for NS1-mediated ubiquitination and subsequent degradation of STAT2^9^; however, it was later shown that RSV NS1 can also cause degradation of OASL, a major cellular ISG^33^, even though STAT2 and OASL are highly dissimilar in sequence.

Our search for potential BC Box Cys residues through mutagenesis failed to identify a functional motif (Supplementary Figs 2 and 3). However, although Cys is the most invariant residue in BC boxes, a few noncanonical BC box motifs have been reported in which Ser or Ala has replaced Cys^39^,^40^,^41^. Of particular mention is the Vif protein of HIV1, which has interesting similarities with NS protein. Both Vif and NS proteins are nonstructural proteins, form dimers, and promote ubiquitination and degradation of host antiviral proteins. Like RSV NS1^9^, Vif assembles an EloBC-Cul-Rbx E3 ligase complex, and the putative NS1 BC box, like that of Vif, contains Ala instead of Cys^39^. The differences include the following: Vif prefers Cul5-Rbx2, RSV NS1 may prefer Cul2-Rbx1; Vif specifically promotes ubiquitination of cytidine deaminase APOBEC3G, a human antiviral protein that causes extensive incorporation error and eventual inhibition of the viral reverse transcriptase^42^,^43^,^44^,^45^,^46^, whereas NS proteins have broader substrate specificity in both RSV^18^,^19^,^21^ and PVM (this study). However, Cul-Rbx pairing of NS can be flexible^9^ and this may in part underlie the ability of NS to accommodate multiple substrates. The crystal structure of the Vif protein in complex with EloBC have suggested that its BC box (including the hydrophobic centre that contains the unique Ala) and Cullin box form a third E3-ligase recruiting site^47^,^48^, indicating a complex multi-subunit interaction, which may also apply to NS. Overall, the increasing degeneracy and complexity of the BC Box highlights the need for an unbiased structural and mutational analysis of the NS proteins of PVM and RSV to identify an apparently unique proteasome recruitment mechanism.

## Methods

### Cells and virus

Immortalized mouse embryonic fibroblast (MEF) cells were obtained from Dr. Ganes C. Sen (Lerner Research Institute, Cleveland Clinic), and grown in monolayer in Dulbecco’s minimum essential media (D-MEM) supplemented with L-glutamine, heat-inactivated fetal bovine serum (10%), penicillin (100 IU/ml) and streptomycin (100 μg/ml). PVM strain J3666 was kindly provided by Dr. Helene Rosenberg (NIH) and was grown in mouse monocyte RAW cell line.

The Tet-inducible ISG cell lines were grown in Tet-free medium as described^34^ and FLAG-ISG were induced by the addition of Tet at 0.2 μg/ml. FLAG-NS1/NS2 plasmids were transfected 18–24 h after Tet addition, and cells were harvested for immunoblot analysis 12–18 h later.

### Antibodies

PVM antibody against Cys-conjugated peptide SQQLNIVDDTPDDDI of the viral N protein sequence (residue 379–393)^49^ was raised in rat commercially (Bio-Synthesis, Lewisville, TX). Primary antibodies used in immunoblot (IB or Western) and immunoprecipitation (IP) were: mouse anti-FLAG (Sigma, SLBF6631/F1804), rabbit anti-FLAG (Sigma, F7425), mouse anti-V5 (Thermo scientific, MA5–15253), rabbit anti-Myc (C-Myc) (Thermo scientific, PA1–981), mouse anti-Ub (Santa cruz, sc-8017), mouse anti-IκBα (Santa cruz, sc-56710), mouse anti-GAPDH (Santa cruz, sc-365062), mouse anti-β actin (Santa cruz, sc-81178), goat anti-RIGI (Santa cruz, sc-48929). The HRP-conjugated secondary antibodies were: goat anti-mouse (Santa cruz, sc-2031), goat anti-rabbit (Santa cruz, sc-2030), and mouse anti-goat (Santa cruz, sc-2354).

### Recombinant plasmids

Codon-optimized NS1, NS2 and P genes of PVM strain J3666 were commercially synthesized by GenScript (Piscataway, NJ) and cloned into the EcoRI-BglII site of the pCAGGS expression vector, such that the proteins were expressed with N-terminal FLAG-tag (DYKDDDDKP)^18^. Like their wild type counterparts, the deletion mutants of NS also carried an N-terminal FLAG tag. Expression plasmids of other recombinant tagged proteins were kind gifts from various investigators: pCAGGS-mSTAT2-FLAG was from Dr. Adolfo Garcia-Sastre (Mount Sinai School of Medicine, NY); Myc-FLAG-mIRF7, Myc-FLAG-mRIG-I, and V5-mIRF3 from Dr. Ganes C. Sen (Lerner Research Institute, Cleveland Clinic, OH). The firefly-Luc reporter plasmids and the control Renilla luciferase plasmid have been described^18^.

### Transfection

All plasmids were prepared using the Midi-Prep kit of Qiagen. MEF cells at 80–90% confluency were transfected using the Lipofectamine LTX with PLUS reagent (Invitrogen/Life Technologies) following the manufacturer’s protocol. In NS transfections, the total amount of transfected plasmids was kept constant in all wells by adding required amounts of vector plasmid (pCAGGS only). Unless otherwise indicated, all transfections were for 24 h.

### Immunoblotting (IB) (Western blotting)

This has been described before^21^. Briefly, cells were harvested at 24 h post-transfection directly in 1× Laemmli sample buffer, and the lysate was heated and sonicated. All samples were then subjected to SDS-PAGE. Except where otherwise mentioned, 4–20% gradient polyacrylamide gels (TGX Stain-Free Gel, Bio-Rad, cat# 4568096) were used. As protein MW marker, we used the Bio-Rad Kaleidoscope ladder (Precision Plus Protein Standards, cat# 1610395), which can be seen in the Supplementary Figures. Proteins were transferred to 0.45 μm PVDF membranes (Immobilon-P, Millipore) and probed using appropriate antibodies. Bands were developed with ECL Prime Western Blotting Detection Reagent (GE Healthcare) and detected in the LI-COR Odyssey Fc imaging system. Where needed, the intensities were quantified with the integrated LI-COR software and normalized against GAPDH or β-actin.

### Immunoprecipitation (IP)

Experiments were performed using protein A-Sepharose (Sigma), as described previously^21^. MEF cells were used and transiently transfected with FLAG-STAT2, Myc-FLAG-RIG-I, V5-IRF3, FLAG-NS. Anti-Myc, anti-V5, and anti-FLAG antibodies were used to pull down the proteins of interest as described in the respective figures. The pellets were analysed by denaturing SDS-PAGE followed by immunoblotting (Western blotting) using anti-FLAG or anti-Ub antibody as appropriate.

### Confocal microscopy

MEF cells (2.0 × 10^5^/well) were plated onto cover glasses in 6-well plates, grown overnight. To detect native RIG-I, cells were infected with RSV at 2 m.o.i for 8 h (which induces RIG-I expression), and then transfected with recombinant FLAG-NS plasmids. For the IRF3 experiments, MEF cells were transfected with recombinant NS plasmids and V5 tagged IRF3. MG132 (Sigma) (10 μM) was added post transfection. Next day, cells were fixed in ice-cold 4% paraformaldehyde for 20 min and permeabilized with PBS containing 0.1% Triton X-100. Fixed cells were blocked in PBS containing 1% BSA for 1 h and labelled for 3 h with the following primary antibodies as appropriate: anti-RIG-I (1:200), mouse anti-V5 (1:200) and rabbit anti-FLAG (1:200). The secondary antibodies were: anti-goat, Alexa Fluor 488-conjugated (1:300); anti-mouse, Alexa Fluor 488-conjugated (1:300); anti-rabbit, Alexa Fluor 594-conjugated. Nuclei were stained with DAPI. Cells were visualized at a 60X magnification in a Nikon A1RSI confocal microscope.

### Dual luciferase assay

These experiments have been described previously^18^. Briefly, appropriate cells were co-transfected with either the empty pCAGGS vector (control) or NS expression plasmid(s) along with IFNβ-promoter-firefly-Luc and pCMV-Renilla-Luc vectors for the indicated duration. Promega’s Dual-Luciferase Reporter Assay System (E1910) was used according to manufacturer’s protocol. Renilla Luc activity served as an internal control for the normalization of the firefly Luc activity. All experiments were performed in triplicates.

### Quantitative RT-PCR analyses

MEF cells were transfected with either of the recombinant plasmids RIGI, IRF3, STAT2, IRF7, and co-transfected with the NS plasmids. Total RNA was isolated, followed by cDNA preparation and qPCR as described^18^.

Real time qRT-PCR was done using the following primers (F = forward; R = reverse): mRIG-I: TGGACAAAAAGGGAAAGTGG (F), TGCTGCACTGAGACGCTATC (R); mIRF3: ACGTGTCAACCTGGAAGAGG (F), GGCACCCAGATGTACGAAGT (R); mIRF7: CCAGTTGATCCGCATAAGGT (F), GAGCCCAGCATTTTCTCTTG (R), mSTAT2: AAACTTCTGAAGGGGGCATT (F); CTTCGGCAAGAACCTGGTAG (R).

### Statistical analysis

Experiments were performed in triplicates (n = 3), and where mentioned, statistical analyses were performed by using GraphPad Prism 5.0 software (San Diego, CA, USA). Mean ± standard deviation (SD) was plotted in all graphs. Student’s t-test and one-way ANOVA was used to analyse all changes. P < 0.05 was considered significant (marked by asterisks in the relevant figures).

## Additional Information

**How to cite this article**: Dhar, J. and Barik, S. Unique nonstructural proteins of Pneumonia Virus of Mice (PVM) promote degradation of interferon (IFN) pathway components and IFN-stimulated gene proteins. *Sci. Rep.* **6**, 38139; doi: 10.1038/srep38139 (2016).

**Publisher's note:** Springer Nature remains neutral with regard to jurisdictional claims in published maps and institutional affiliations.

## Supplementary Material

Supplementary Material
