# Supplementary material for: Unique nonstructural proteins of Pneumonia Virus of Mice (PVM) promote degradation of interferon (IFN) pathway components and IFN-stimulated gene proteins
Source: Sci Rep. 2016 Dec 1;6:38139. doi: 10.1038/srep38139 (PMC5131486; doi:10.1038/srep38139)

Supplementary material

Unique nonstructural proteins of Pneumonia Virus of Mice (PVM) promote degradation of interferon (IFN) pathway components and IFN-stimulated gene proteins

Jayeeta Dhar and Sailen Barik*

Department of Biological, Geological and Environmental Sciences, and Centre for Gene Regulation in Health and Disease, Cleveland State University, 2121 Euclid Avenue, Cleveland, Ohio 44115

*Corresponding author:
Phone: 216-523-7326; Fax: 216-687-5549
E-mail: s.barik@csuohio.edu

Supplementary Figure 1

a

```
PVM-NS1    MGCNVMMELDYG-------------------G-------RAAWLAFHITNFDRSDLETILRGARVCNTWQDQRLSVYLVG   54
RSV-NS1    MGSNSLSMIKVRLQNLFDNDEVALLKITCYTDKLIHLTNALAKAVIHTIKLNGIVFVHVITSSDICPNNNI-------VV   73
           **.* :  :.                               *  .:*  :::   :  :: .: :* . :        *

PVM-NS1    RDCNLLRPFVQAAKFIHNTRRGQTLTHWFTKNIVFSSTGQETEPPIDPTCELLVELISG---------------------  113
RSV-NS1    KSNFTTMPALQNGGYI-----------WEMMELT---HCSQPNGLIDDNCEIKFSKKLSDSTMTNYMNQLSELLGFDLNP  139
           :.     *.:* . :*           *   ::.     . ::  ** .**: ..   .
```

b

```
PVM-NS1    MGCNVMMELDYGGRAAWLAFHITNFDRSDLETILRGARVCNTWQDQRLSVYLVGRDCNLLRPFVQAAKFIHNTR------   74
RSV-NS2    ------MDTTHNDT-TPQRLMITDMRPLSLETTITSLTR--D-IITHRFIYLINHECIVRKLDERQATFTFLVNYEMKLL   70
                 *:  :    :   : **::   .*** : .          :  :**: ::* : :   : *.* . ..

PVM-NS1      --RGQT----LTHWFT------KNIVFSSTGQETEPPIDPTCELLVELISG-----  113
RSV-NS2      HKVGSTKYKKYTEYNTKYGTFPMPIFINHDGFLECIGIKPTKH--TPIIYKYDLNP  124
                *.*     *.: *        *.:.  *      *.**    . :*
```

c

```
PVM-NS2    MSTAMNKFTQTISKPATILNISDSEESGDEAGVGKVSRTTQSSERWLDLLIEKFQPS-----LQNITR-YINWNFIRICN   74
RSV-NS2    MDT------------------------------------THNDTTPQRLMITDMRPLSLETTITSLTRDIITHRFIYLIN   44
           *.*                                    *:..     *:* .::*      : .:**  *. .** : *

PVM-NS2    DRLKKEKMGYIEA-KQYVEDMAWMVIASEADSIEWKCIRRQEKVTGVKYPKFFFVQHKEDWIECTGCIPYPGHDLIYDED  153
RSV-NS2    HECIVRKLDERQATFTFLVNYE-MKLLHKVGSTKYKKYTEYNTK-YGTFPMPIF-INHDGFLECIGIKPTKHTPIIYKYD  121
           ..   .*:   :*   :: :   * :  :. * ::*   . :.    .:*  :*  .:: ::** *  *     :**. *

PVM-NS2    DDD  156
RSV-NS2    LNP  124
            :
```

d

```
PVM-NS2    MSTAMNKFTQTISKPATILNISDSEESGDEAGVGKVSRTTQSSERWLDLLIEKFQPSLQNITRYIN--------------   66
RSV-NS1    ------------------------------MGSNSLSMIK----VRLQNLFDNDEVALLKITCYTDKLIHLTNALAKAVI   46
                                          *  .:*  .      *: *::: : :* : ** * :

PVM-NS2    -------WNF------IRICNDRLKKE-----KMGYIEAKQYVEDMAWMVIASEA-DSIEWKCIRRQ-EKVTGVKYPKFF  126
RSV-NS1    HTIKLNGIVFVHVITSSDICPNNNIVVKSNFTTMPVLQNGGYIWEMMELTHCSQPNGLIDDNCEIKFSKKLSDSTMTNYM  126
                    *        ** :.         .*  ::   *: :*  :. .*:    *: :*  :  :*::  .  :::

PVM-NS2    FVQHKEDWIECTGCIPYPGHDLIYDEDDDD  156
RSV-NS1    -----NQLSELLGFDLNP------------  139
                ::  *  *    *
```

Legend of Supplementary Figure 1. Dissimilar sequences of the nonstructural proteins of RSV and PVM. These pairwise alignments, performed with Clustal Omega[1], actually show the *lack* of any significant similarity between the NS proteins of the two viruses. We tested all four combinations, as we did not know *a priori* which NS protein of PVM could be homologous to which NS protein of RSV, if at all. The hypothetical SOCS BC Box sequence of RSV NS1 protein, suggested previously[2], is underlined in panel A, with the conserved Leu and Cys residues highlighted. However, since this sequence had no discernible similarity with any PVM NS, we conjectured a few other possible SOCS BC Box motifs (boxed) to further illustrate that they too had only distant similarity with BC Box, if at all. The GenBank Accession numbers of the sequences are: AAW02832.1 (NS1, PVM strain J3366); YP_173325.1 (NS2, PVM strain J3666); AAA79091.1 (NS1, RSV Long); AAA79090.1 (NS2, RSV Long).

Supplementary Figure 2

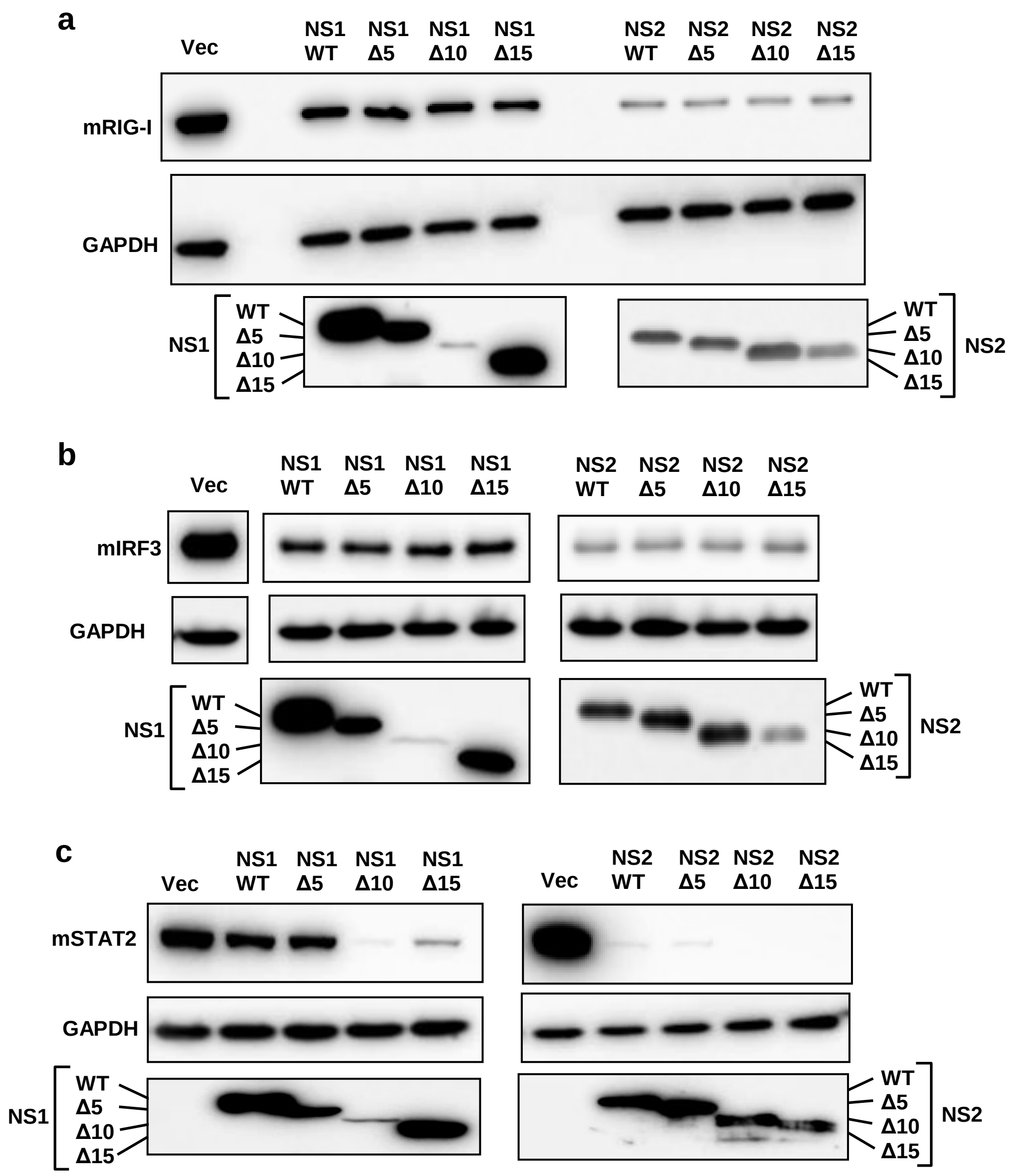

Legend of Supplementary Figure 2. Functional studies of PVM NS C-terminal deletions. The NS1 and NS2 C-terminal deletions (Δ5, Δ10, Δ15) were constructed as described previously for RSV[3]. Like their wild type counterparts, the deletions carried an N-terminal FLAG tag (DYKDDDDKP). The degradative activity of the deletions were tested against (a) RIG-I, (b) IRF3 and (c) STAT2, by co-transfection of the recombinant plasmids into MEF cells, followed by immunoblot. In each panel, expression of the NS proteins was also monitored, and GAPDH was the loading control. Note that all deletions were as active as their wild type (WT) counterparts. The NS1Δ10 deletion actually appeared more active than the wild type even though it was expressed in much smaller amounts; the reason for this remains unknown. (Original uncropped panels of all blots are presented at the end of this file).

Supplementary Figure 3

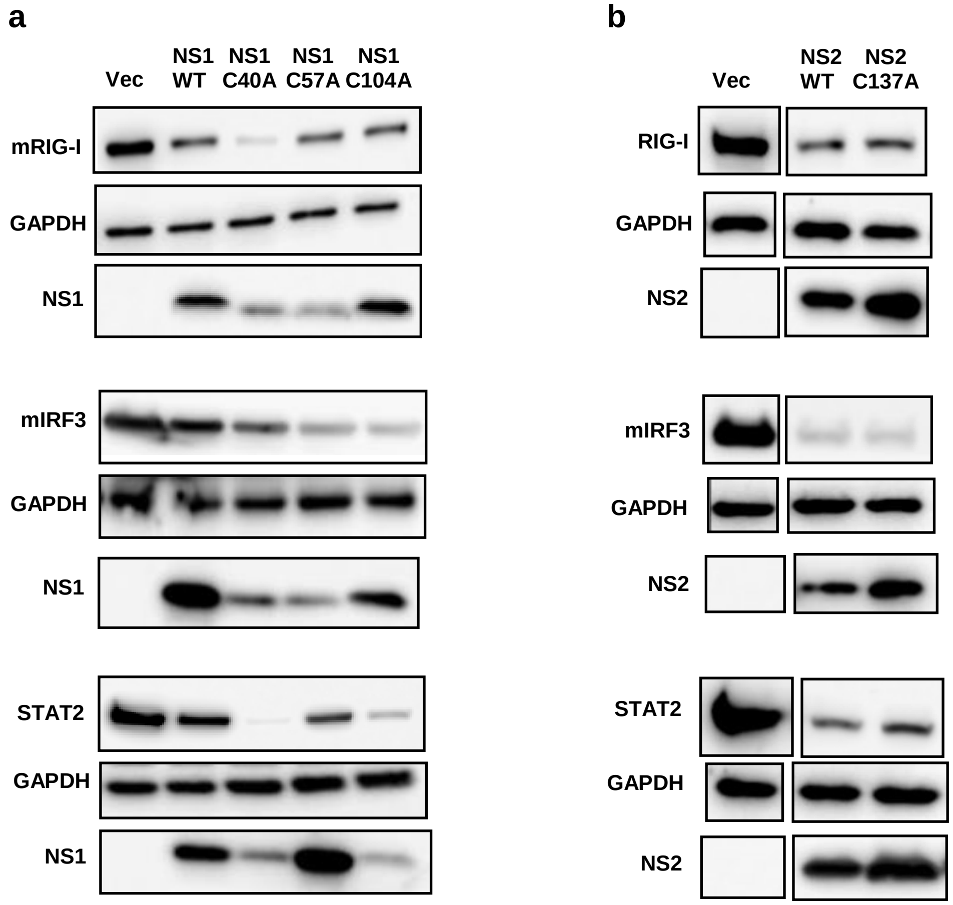

Legend of Supplementary Figure 3. Functional studies of selected Cys mutants of PVM NS. The indicated Cys residues, some belonging to presumptive SOCS Box motifs (Supplementary Fig. 1), were mutated to Ala by using QuikChange II Site-Directed Mutagenesis Kit (Agilent). The recombinants were tested for their degradative activity in transiently transfected MEF cells. A total of five mutants (four NS1 and one NS2) were tested against RIG-I, IRF3 and STAT2 as shown and none of the mutations abrogated NS activity. GAPDH served as loading control. (Original uncropped panels of all blots are presented at the end of this file).

References to Supplementary Material:

1. Sievers, F. *et al.* Fast, scalable generation of high-quality protein multiple sequence alignments using Clustal Omega. *Mol. Syst. Biol.* **7,** 539 (2011).
2. Elliott, J. *et al.* Respiratory syncytial virus NS1 protein degrades STAT2 by using the Elongin-Cullin E3 ligase. *J. Virol.* **81,** 3428-3436 (2007).
3. Swedan, S., Musiyenko, A. & Barik, S. Respiratory syncytial virus nonstructural proteins decrease levels of multiple members of the cellular interferon pathways. *J. Virol.* **83,** 9682-9693 (2009).

Selected ORIGINAL files for the corresponding Figures

Fig. 1

(a)

RIG-I and actin panels

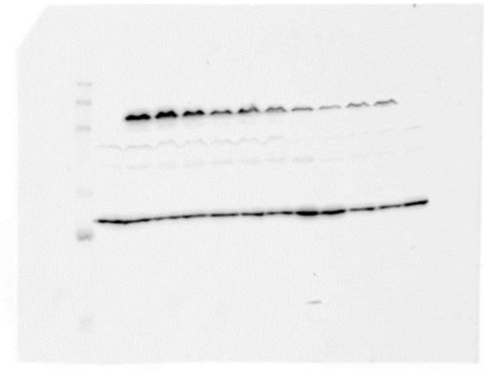

NS panel

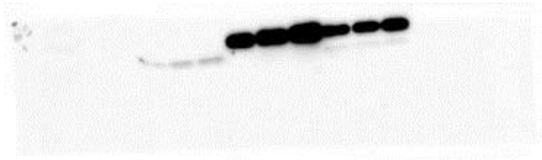

(b)

mRIG-I panel (straightened; and the last lane was removed because it was an extra sample).

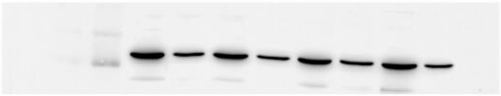

NS panel (the first four blank lanes were extra untransfected samples, cropped out for submission).

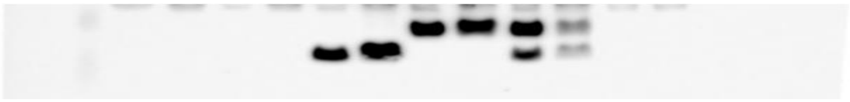

GAPDH panel, run on a different membrane; only the first six lanes were taken; the others were irrelevant and therefore cropped out.

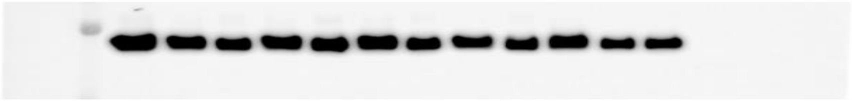

(c) IP panel

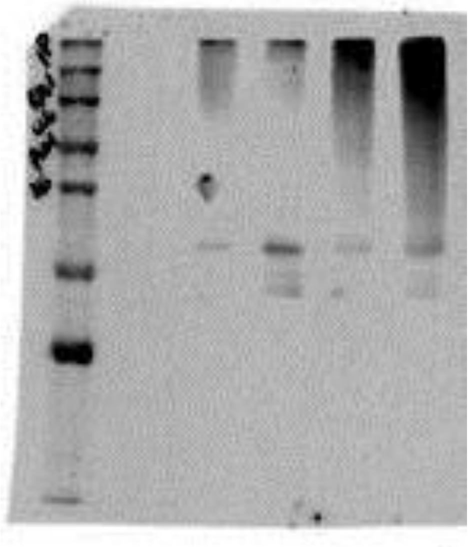

<u>**Fig. 2**</u>

(a)

mIRF3 panel

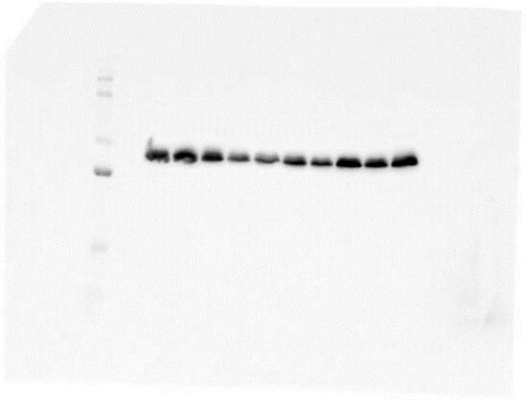

NS panel (the last three lanes were loaded in the reverse order by mistake. This was corrected for submission, and borders were added).

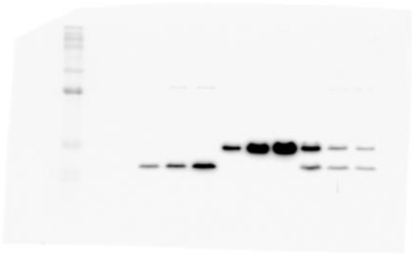

GAPDH panel

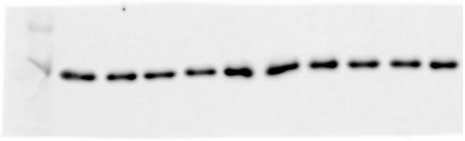

(b)

mIRF3 panel (the first two irrelevant sample lanes were cropped out)

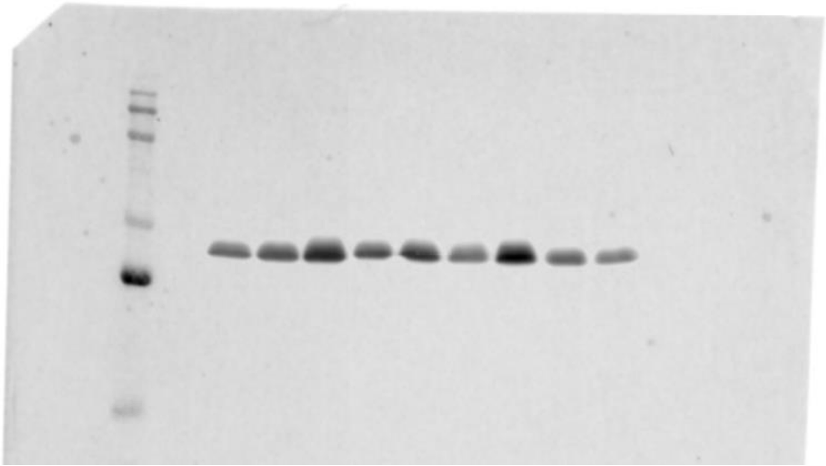

NS panel: separate gel was run; empty lanes were later removed; one of them had sample leak form the neighbouring lane; shown by boxes in the submitted Fig.

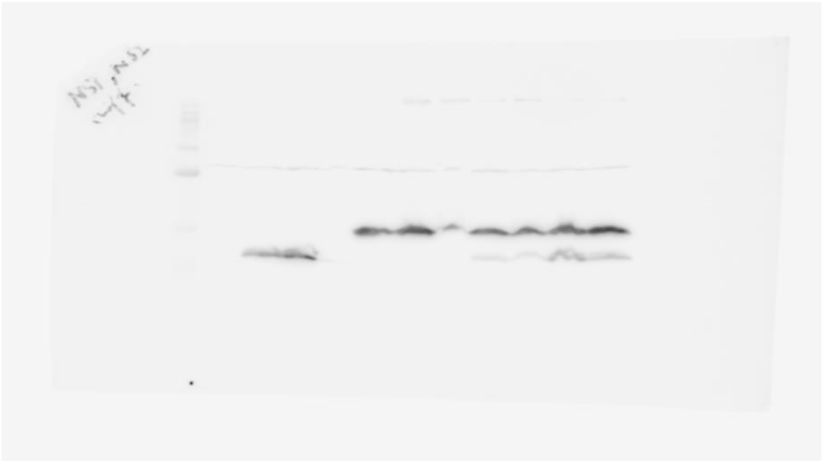

GAPDH panel: the first three and the last two were for some other samples; they were removed for submission.

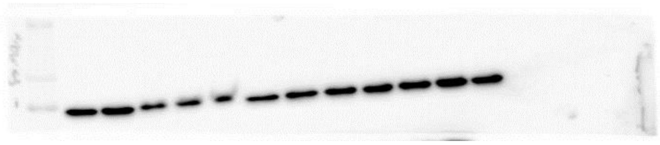

(c)

IP panel: unloaded lanes were removed for submitted Fig.

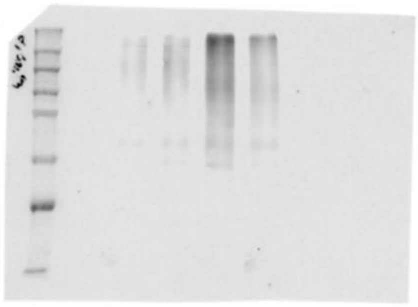

Fig. 3

GAPDH and Actin panels:

Actin (lower band) was taken from the longer exposure; but the mIRF7 panel was overexposed, so it was taken from the shorter exposure.

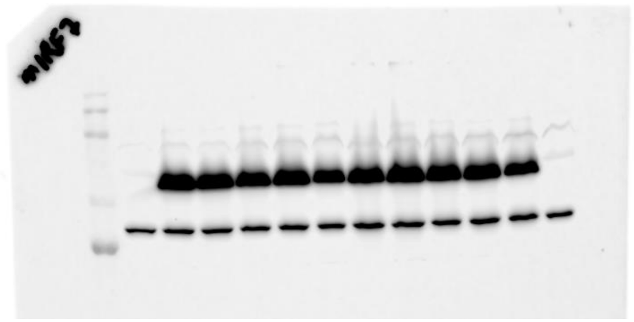

Longer exposure (for Actin)

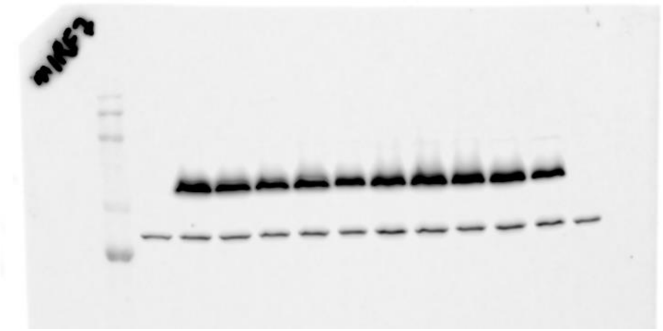

Shorter exposure of the same blot (for mIRF7)

NS panel; the blank lanes were removed.

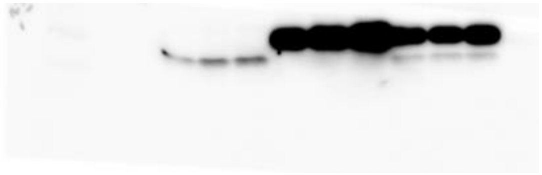

Fig. 4

(a)

mSTAT2, actin panels (top) and NS panel (bottom): The first lane was untransfected control; it was always removed in the submitted Figs. for consistency.

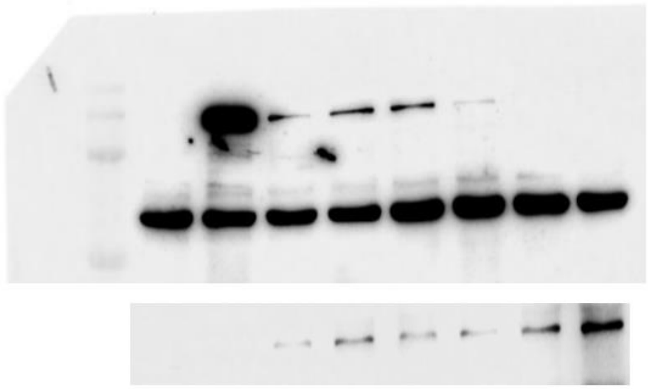

(b)

All panels from the same blot (using mixture of antibodies), different exposures

mSTAT2 (top band) and actin (3rd band from top) taken from this exposure:

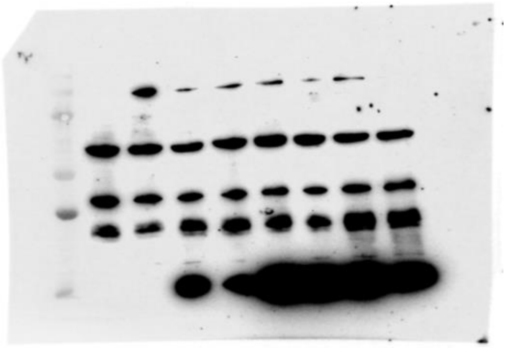

NS (lowest band) taken from this exposure:

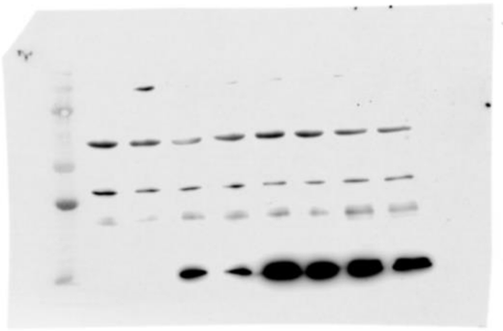

(c)

mSTAT2 and actin panels (only the relevant lanes taken for Fig.)

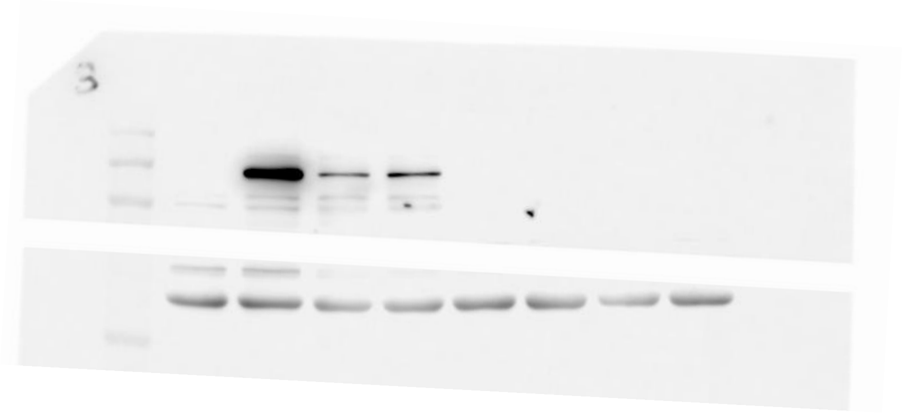

NS panel

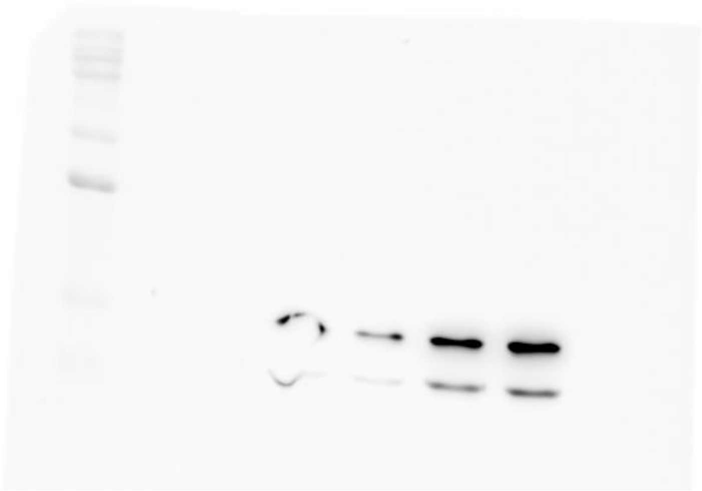

(d)

mSTAT2 and NS panels: the first two lanes and the last lane were irrelevant and removed in the submitted Fig.

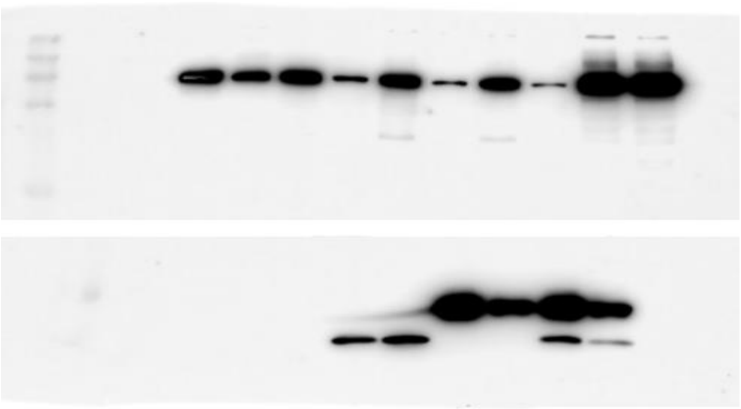

Actin panel: the lowest band is actin; others are nonspecific; only the first 7 lanes are relevant.

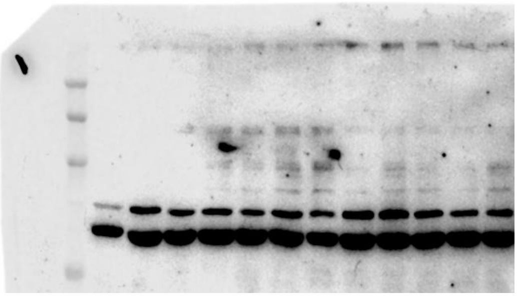

(e)

IP panel

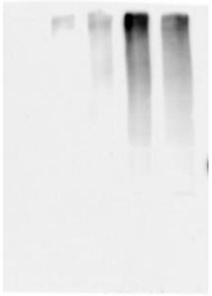

<u>Fig. 5 is a virus blot, same as the raw data</u>

<u>Fig. 6 is luciferase assay</u>

<u>Fig. 7</u> All panels are shown below. In general, all blots show marker protein ladder on the left , and extra lanes were sometimes removed. Comparison with the submitted Figure will make the submitted lanes obvious. For GAPDH, only the last four lanes were taken since we decided to not present the first sample (no ISG) to save space.

(a)

<u>TRAFD1 data:</u>

TRAFD1 NS1/NS2 GAPDH

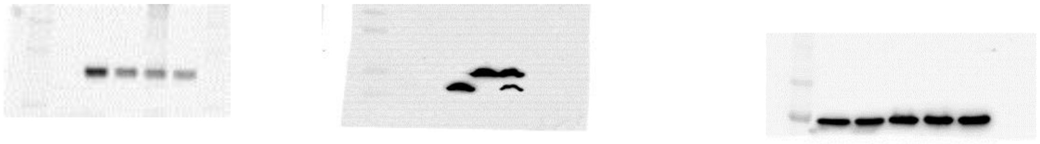

IFITM1 data:

Single membrane; IFITM1(upper band) and NS1/NS2 (the two lower bands)

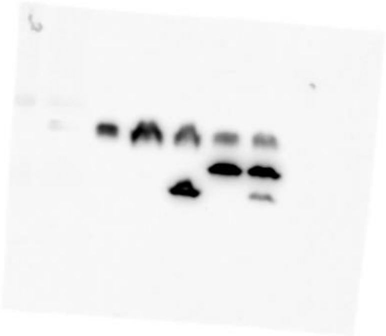

GAPDH

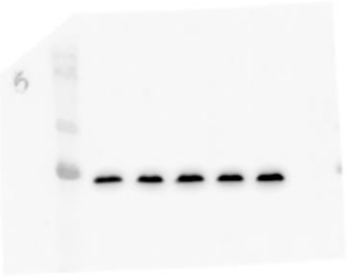

ISG20 data:

ISG20 (cropped out to separate from the much darker NS bands; see the same NS bands in underexposure below)

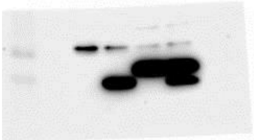

GAPDH

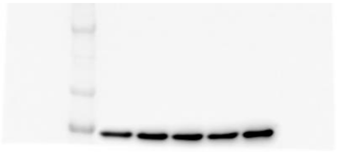

NS1/NS2

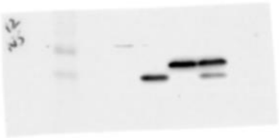

IDO data:

IDO

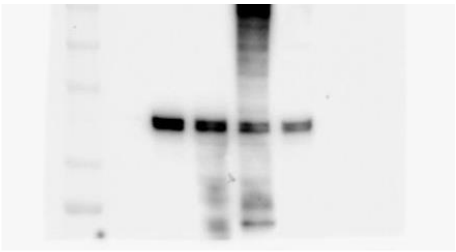

NS1/NS2

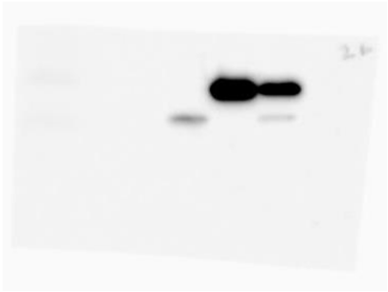

GAPDH

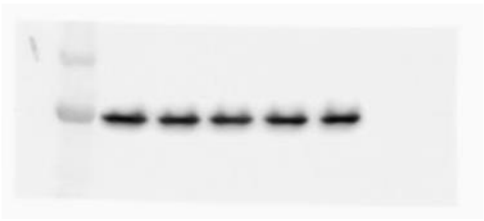

IFIT3 data:

IFIT3

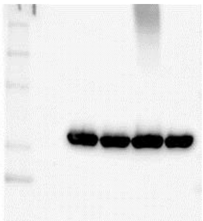

NS1/NS2

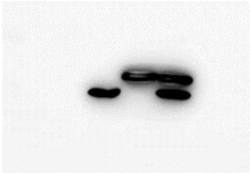

GAPDH (lower band)
(Upper band is carryover ECL of IFIT3)

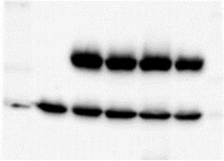

Viperin data:

Viperin

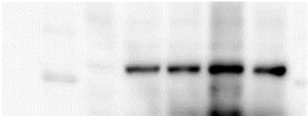

NS1/NS2

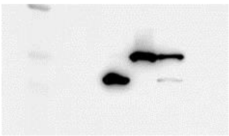

GAPDH

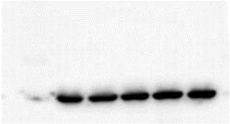

AKT data:

AKT (top bands)

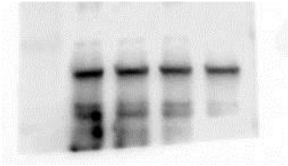

NS1/NS2

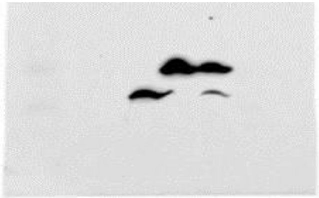

GAPDH (lower band)

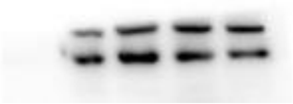

CIITA data:

CIITA

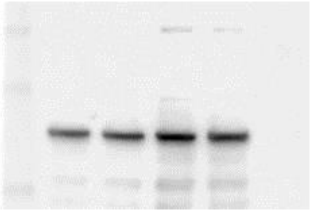

NS1/NS2

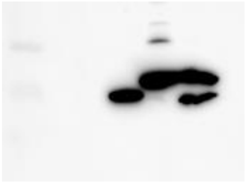

GAPDH (lower band)

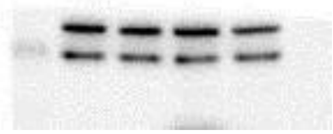

S6K data:

S6K

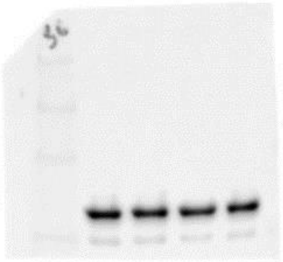

NS1/NS2

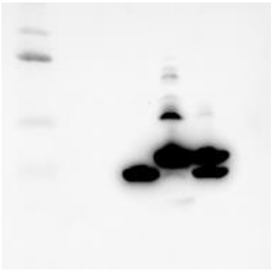

GAPDH (lower band)

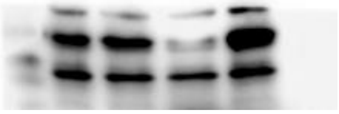

(b)

RIG-I data:

RIG-I and PVM-P

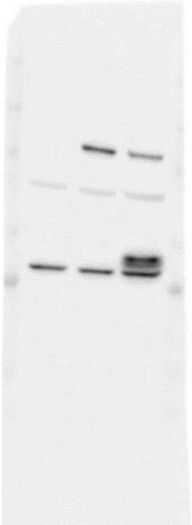

GAPDH

IRF3 data:

IRF3

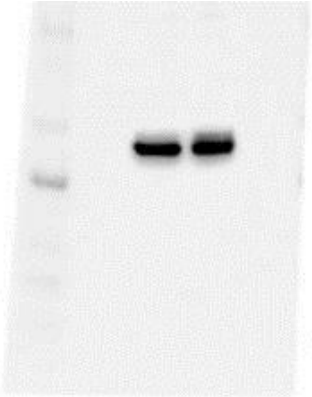

PVM-P

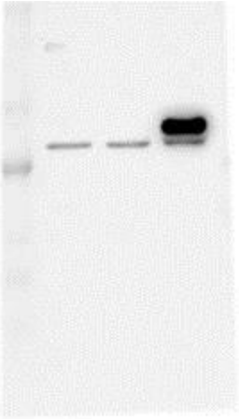

GAPDH

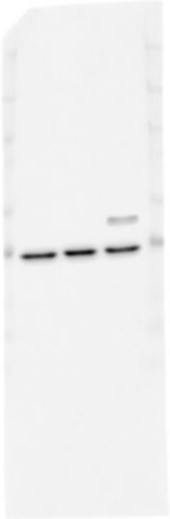

TRAFD1 data:

TRAFD1

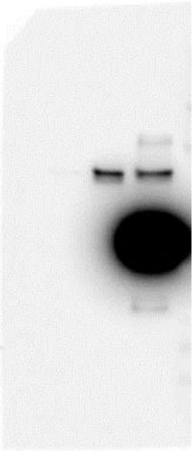

PVM-P

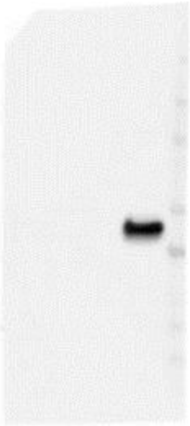

GAPDH (only the last two lanes taken)

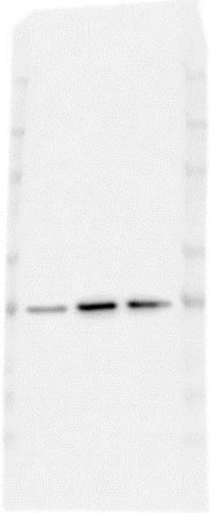

Fig. 8: AB are microscopic images; C is an intact blot

Supplementary Figures

Suppl Fig. 2

(a)

NS1 and NS2 samples were run separately, and as such, with border added in the submitted Fig.

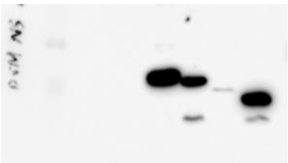

(b)

mIRF3 panel; blank (gap) lanes removed, indicated by boxes in the Fig.

Also, the NS1-transfected and NS2-transfected sets were inadvertently loaded in the reverse order; therefore, they were rearranged for proper order of presentation, matching with other Figs.

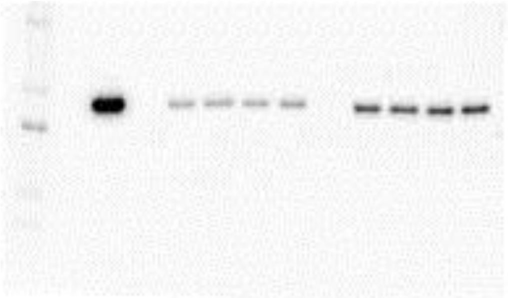

GAPDH panel: Extra lane on left (not transfected with mRF3 plasmid) was removed.

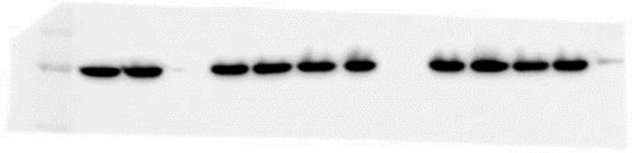

NS1 and NS2 panels

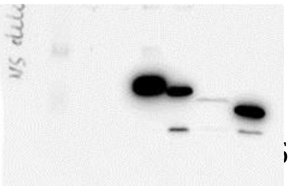

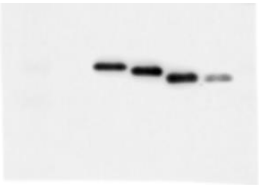

(c)

mSTAT2 panel: upper band; the lower band is background

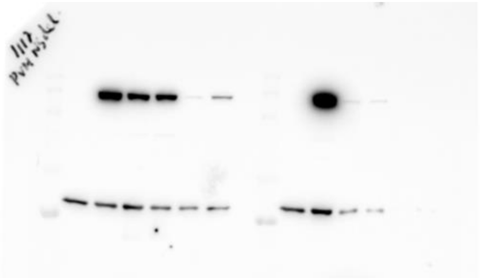

GAPDH panels: First lane in the left blot and last lane in the right blot were discarded.

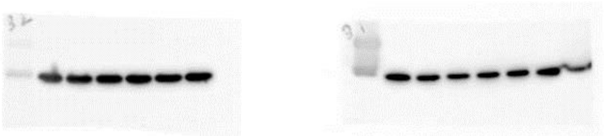

NS panels (lower bands); the higher bands are background and were removed.

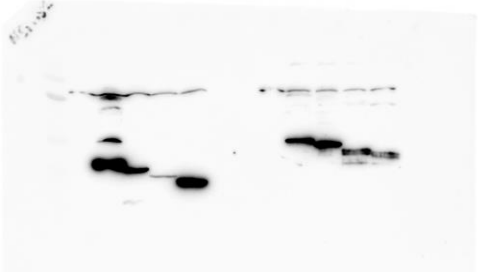

Suppl Fig. 3

(a)

RIG-I panel

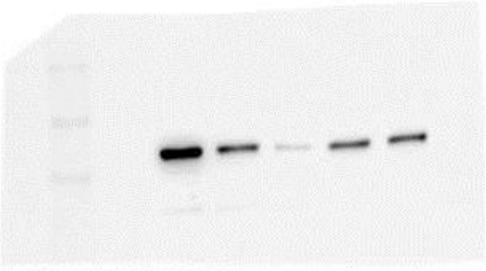

GAPDH

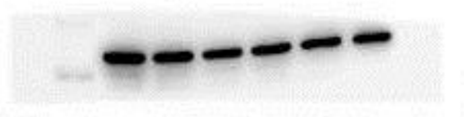

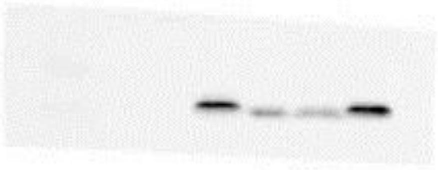

NS1 panel

mIRF3 (IRF3-untransfected first lane removed)

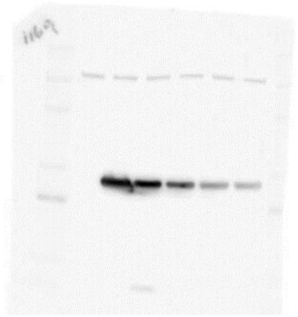

GAPDH: different gel run (last lane discarded)

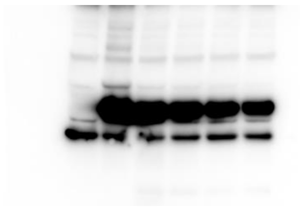

NS1 panel

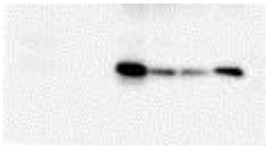

STAT2 panel

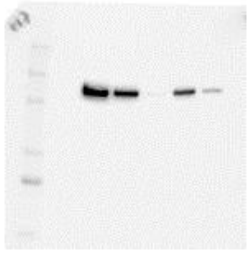

GAPDH: Lower band (first lane not relevant and discarded)

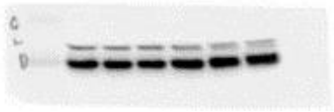

NS1 panel:

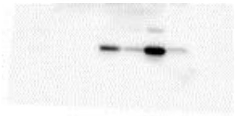

(b)

RIG-I (top) and GAPDH (bottom): In both blots, only lanes 1, 3, 4 were taken; others were irrelevant.

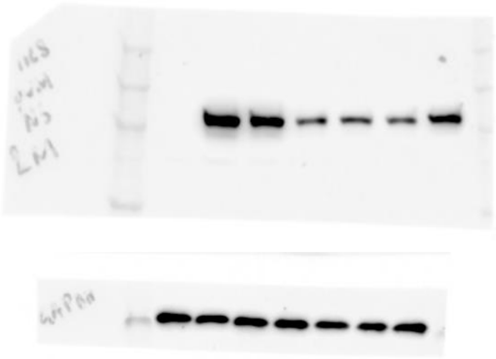

NS2 panel: only lanes 1, 3, 4 were taken for relevance

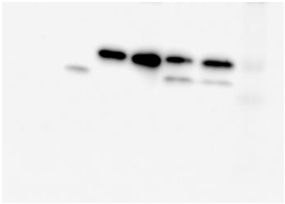

mIRF3: only lanes 1, 3, 4 were taken for relevance

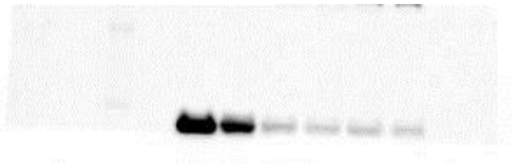

GAPDH: only lanes 1, 3, 4 taken for relevance

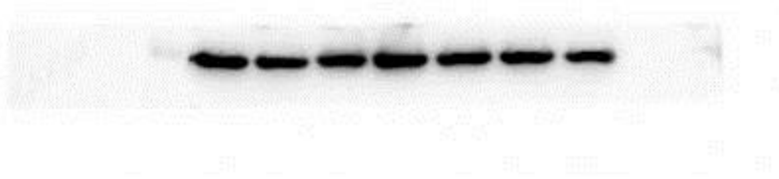

NS2 panel: only lanes 1, 4, 5 are relevant; the lower band is non-specific

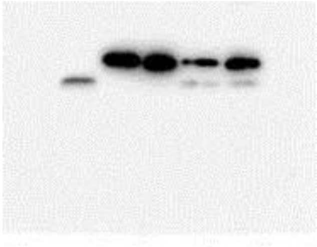

STAT2: only lanes 1, 3, 4 are relevant

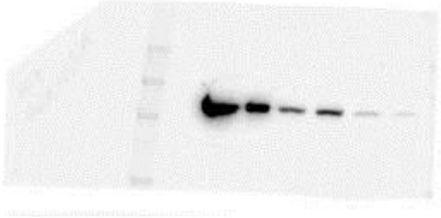

GAPDH: only lanes 1, 3, 4 are relevant.

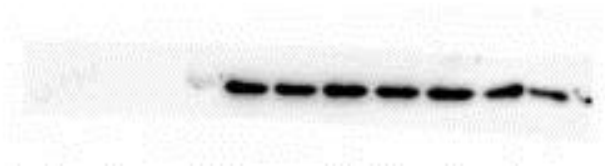

NS2: only three lanes are relevant.

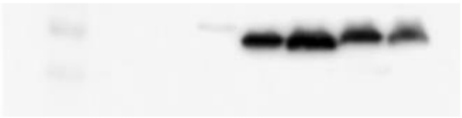

Supplement: Supplementary Material [file srep38139-s1.pdf]
